# What you see may not be what you get! Simulate towards effective planning of pediatric intensive care unit

**DOI:** 10.3389/fped.2022.903601

**Published:** 2022-09-06

**Authors:** Sujatha Thyagarajan, Sindhu Malvel Gowda, Chetan Ginigeri, S. Anupama, R. Chinnadurai

**Affiliations:** ^1^Department of Pediatric Intensive Care Unit, Aster RV Hospital, Bengaluru, India; ^2^Department of Intensive Care Unit, Aster RV Hospital, Bengaluru, India

**Keywords:** simulation, planning, PICU design, *in situ* simulation, low-cost, low resource

## Abstract

**Aims and objectives:**

This study aimed to describe the application of low-cost inter-professional simulation over 4 phases in identifying structural and design issues, latent safety threats as well as test systems, processes, including facilitated team training during the design of a new pediatric intensive care unit (PICU).

**Materials and methods:**

The four-phase inter-professional simulation sessions involving clinical and non-clinical teams were conducted over a 3-month period in a corporate hospital during the designing of a new PICU. Low-cost resources, such as floor tapes, low-tech manikins, reused sterilized consumables, and actual patient beds and equipment, were used for the *in situ* simulation sessions. A plus-delta method of debriefing was done, and changes agreed on consensus were implemented after each simulated session.

**Results:**

There were 10 simulation sessions conducted over 4 phases during the 3-month period of designing the PICU. The participants included 10 doctors from PICU and adult critical care, 25 critical care nurses, 12 members from the project team, and 2 hospital administrators in various combinations. The first phase led to the re-design of workspace and clinical areas for better space utilization. The second phase required further revision to facilitate better mobility and facilities. In the third phase, the number of beds was reduced to 6 beds following the simulated drills involving the actual placement of patient cots and equipment. The fourth phase had thematic 5 simulated exercises involving the newly recruited clinical teams that enabled the identification of systems and process issues. Cognitive aids and video orientation of the setup, team training, and human factors training were addressed, and the unit was open for patient care in a week.

**Conclusion:**

A phased inter-professional simulation exercise with low-cost resources can enable the identification of structural challenges, design issues, latent safety threats, test systems, processes, patient flow, and facilitated team training during the design of a new PICU. Further studies are needed to understand the generalization of the study findings into designing PICU.

## Introduction

The pediatric intensive care unit (PICU) is a highly specialized critical care area for children within hospital premises. Safety is a key feature to ensure quality care for critically ill children, and hence, designing a unit that is user-friendly for clinical teams and addressing the key safety features are vital at the outset. There are guidelines available for setting up intensive care units, which focus on the design, facilities, equipment, and personnel (1, 2). However, the consistency of application and the benefits or risks of implementing such guidelines are hardly studied. Often, clinical teams are recruited after the unit is constructed and the key input of clinicians or end users toward the design of the critical care area is missed. There is a need to identify the strategies that enable the engagement of clinical teams with the project team for safe designing of critical areas.

Simulation as a methodology has been applied in the context of setting up the new clinical environment, testing processes, and systems, team training, and identification of latent safety threats in healthcare settings (3–5). The application of SIMtest in the design of PICU as a teamwork among the projects department, administrators, and clinical teams is described by the Barcelona team (3). However, there is hardly any experience reported in setting up a PICU in India by applying simulation as a methodology.

In this article, we describe a 4-phase simulation-based study similar to SIMtest with the unique application of low-cost simulation methodology in setting up a PICU in the Indian context. The main objectives of the first phase in the pre-construction stage were to identify structural challenges and phase two to determine the design issues during the setting up of a tertiary PICU *via* low-cost simulation methods. The objectives of the subsequent 2 phases of *in situ* simulated exercises in the post-construction stage were to test the facility for functionality in phase 3 followed by testing for safety and preparedness, identify latent safety threats, test systems, and processes and team training for opening the unit for clinical care in phase 4 of the study.

## Materials and methods

### Setting

A corporate hospital with a plan to design a PICU on the 7th floor of the hospital is adjacent to the existing adult critical care facility.

### Participants

Clinicians were predominantly senior adult and pediatric critical care consultants, senior critical care nursing team, the newly employed pediatric critical care team of doctors and nurses, the project team of structural and civil engineers, and senior hospital administrators.

The clinical lead for the proposed PICU led the simulation sessions *via* low-cost measures to evaluate and debrief in a phasic manner. The goals and objectives of each phase were defined, a plus and delta method of debriefing was done at the end of the simulated drill, and the changes were implemented accordingly over all the phases. It was mandatory for all the senior team members to be present for all 4 phases of implementation.

A 4-phase simulation study for designing a new PICU over a 3-month period was implemented. At least one simulated session for each phase was envisioned and add-on sessions were planned mainly to test the changes suggested. The participants required were determined by the objectives of the session and invited accordingly to maximize the outcomes per session (refer to Table 1).

**TABLE 1:** Four phases of simulation in the design of PICU.

Phases	Objective	Simulation methods	Participants
1st	Review of floor plans versus actual area	*In situ* simulation with a field visit and application of floor tapes.	Doctors, Civil project team of engineers, Administrators
2nd	Review of the functionality of areas allotted in the floor plan.	*In situ* simulation by area measurements and floor tape application	Senior doctors and nurses Civil project team of engineers, Administrators
3rd	Review of the functionality of the clinical area.	*In situ* simulation by mobilizing actual medical equipment and beds in each allotted space.	Senior doctors and nurses, Civil project team of engineers, Administrators
4th	Review and check the preparedness of the current setup of clinical area for patient admission	Thematic simulation scenarios to check – patient flow, emergencies in PICU, PICU procedures, communication, and operational issues *via in situ* simulation and low-cost resources	PICU team – Doctors, Nurses

#### Phase 1

The key focus during this phase would be the structural challenges of designing the unit. Review of floor plans versus actual area proposed for the design of PICU through field visit. Floor tapes to simulate the proposed plans were applied. Simulation drill was led by the clinical team with experience in simulation and debriefing. A plus and delta method of debriefing was planned and action plans were outlined with key responsibilities and persons for implementation.

#### Phase 2

The main objective during this phase would be to identify design challenges. Review of floor plan versus actual area, application of floor tapes to simulate the proposed design of the PICU to check the functionality of the area, such as nursing station, clean/dirty utility areas, mobilization of equipment, appropriateness of electrical and plumbing requirements, and gas supply. Simulation exercise comprised of inter-disciplinary teams mainly nursing, housekeeping, maintenance, the project team of engineers.

#### Phase 3

The main purpose of this phase would be to determine the functionality issues during the post-construction phase of setting up the PICU. Review of the PICU area after basic construction was completed to simulate the actual clinical area by the placement of all the beds and the equipment to check the functionality of the unit. Simulation exercise involved setting up each of the bed spaces with beds, monitors, ventilators, infusion pumps, and mobility of the patient in the designated clinical areas. Clinical teams and project teams participated in this exercise. A plus and delta method of debriefing was led by the critical care team. Further exercises to ensure the final implementation of the plan were done as per the debriefing action points.

#### Phase 4

The key objective of this phase of the study was to determine the system and team preparedness before opening up to direct patient care. Thematic *in situ* simulation exercise with the newly appointed pediatric critical care team was done to check the preparedness of the unit and the process and system checks after setting up the unit for patient care. Low-tech pediatric and adult manikins and airway manikins were used for the scenarios. SimpL app (6), a simulation training application for healthcare professionals, was used to simulate the bedside monitor *via* tablet to demonstrate the changes in trends of vitals. The drugs and the consumables used for the scenarios were the disposables that were expired or sterilized after patient use from the adult critical care unit. Embedded participants were carefully selected from the hospital team for each of the scenarios. Daily simulation exercises with various combinations of PICU team members were planned to identify and rectify the issues in the newly formed unit. A plus and delta method of debriefing was led by the lead PICU consultant to implement action plans of issues identified during simulation. The institutional ethics committee exempted the study from approval since the study did not involve patients or data of patients. All participants of the study consented to the sharing of photographs for publication.

## Results

There were 10 simulation sessions conducted over 4 phases during the 3-month period of designing the PICU, which is summarized in Table 1. The participants included 10 doctors from PICU and adult critical care, 25 critical care nurses, 12 members from the project team, and 2 hospital administrators in various combinations who participated in the simulation drill. The key debriefing points and the outcomes are summarized in Table 2.

**TABLE 2:** Key debriefing points during each phase of simulation and outcomes.

Phases	Plus	Delta	Learning points	Outcomes
Phase 1 (1 session)	Consensus of all teams about the suitability of the proposed area for PICU An acute care area (PICU) can be designed given the area suggested. Does require some structural modifications or investment	Pre-set gas pipes, plumbing and electric sockets have to be considered during drafting of floor plans The difficulty for clinical teams to visualize the space as PICU (drawings versus empty space) The designing was challenging as the space had to be modified to build PICU than a pre-planned space during building construction	Better to plan acute patient care areas during initial planning rather than add-on designing. Field visit by the project team and understanding the layout – electrical, gas, and plumbing are important during drafting of the floor plans Discuss priorities of clinical teams for patient safety Open discussions between teams are vital for meaningful safety measures	Changes were made in the initial floor plan with re-design of the workspace and clinical areas based on the gas and plumbing arrangements.
Phase 2 (1 session)	Floor tapes application of modified plans helped in understanding the challenges better Nursing team’s input of priorities was very useful Administrators were very open to modified planning and focused on safety aspects	Practicalities of locations of storage, nursing bay, and utility areas to be planned considering the bed movements and the lighting for better care.	Field visit is important to understand the priorities of clinicians and project team Important to focus on storage, utility area, nursing bay during planning Nursing inputs vital at early stages Redo of the simulation of the agreed plan was helpful to get a better consensus	Re-modification in the floor plan with floor tapes was made. Areas were reviewed and actual visualization of bed spaces, utility area, storage areas, and the nursing bay was modified.
Phase 3 (1 session)	Able to remodify the bed spaces after placing equipment and the functionality was approved by clinical teams Consensus to reduce the number of beds achieved between clinical and non-clinical teams	Space for equipment movements and care was underestimated during floor tapes application exercise Could consider cardboard simulation for more realism	Functionality achieved and important to do the equipment placement and simulation drill Able to achieve the required outcomes of safe designing with floor tapes and equipment and clinical team simulation as a low-cost alternative	Space allocation better suited for 6 beds than the initial plan for 8 beds. Floor plans were re-designed and implemented for 6 beds.
Phase 4 (i)Patient flow (2 sessions)	Use of low-cost *in situ* simulation methods and equipment for the simulation sessions *In situ* simulation with various team member combinations and conducting simulation drills during day and night Bridging of gaps with each sim drill	Situational awareness – familiarity with team members, environment and processes Communication between team members and with the rest of the hospital team members IT issues leading to delays	Video orientation of the PICU team to show the processes and the equipment in the PICU Ensuring backup IT systems and hence 2 Computers Training of nurses for new IT systems to ensure smooth indenting processes	Implemented video orientation for staff. Backup IT systems placed in PICU Nursing training of IT systems completed and competency checked and signed off.
(ii) Emergencies in PICU (2 sessions)	Use of low-cost *in situ* simulation Familiarization of team members to manage the emergencies anticipated and gaining confidence Sharing of lessons within the WhatsApp group Code blue and difficult airway processes reinforced	Human factors – situation awareness, communication and confidence Processes for restricted drugs maintenance and crash cart checklist	Video orientation Sharing of lessons within the WhatsApp group Reinforcement of learning through regular simulation sessions	Creation of WhatsApp group of all team members and lessons learnt shared by participating team for multiplier effect. SOP shared within the group for all to reinforce Nurse in charge supervised real-time all the nurses in the PICU processes and signed them off for competency
(iii) PICU procedures (1 session)	Familiarization of available consumables, inventory maintenance, troubleshooting for failed procedures, escalation matrix	Lack of timely management of equipment by nursing staff	Reinforced biomedical training for specific team members Nano-simulation drills for arranging intubation equipment and ongoing training	Biomedical training completion logbook completed for all the nursing and medical staff Nano-simulation training for all nurses about setting up for intubation and recording the time taken and demonstrating the improvements.
(iv) Communication (1 session)	Familiarization of video counseling processes, checks of overhead speakers Familiarization of handling of difficult families and escalation matrix	Need for telephone lines and smart phone as backup Need of extra speakers in the corridor and counseling room	Smartphones and extra telephone lines placed Speaker checks timetable daily with soft music	Smartphone access and extra overhead speaker implemented. Maintenance teams roster to do overhead speaker checks daily implemented.
(v) Operational issues (1 session)	Familiarization of different codes to enter on the Electronic health records for billing Familiarization of security escalation matrix Familiarization of fire safety measures	Need to add extra codes suited for pediatric packages Security responses to the new clinical area – requires training	Pediatric packages codes to facilitate care for poor patients Training of security teams	Pediatric care billing packages designed and implemented, with competency of relevant staffing logged. Security staff competency logged in.

### Phase 1

During phase 1 of the field visit, the clinical team, hospital administrators, and the project team participated in this exercise. A plus and delta debriefing was done and an action plan was formulated. Changes in the initial floor plans were modified, such as re-design of the workspace and clinical areas compared to the original draft, which would be more suitable for plumbing, electrical sockets, gas pipes, and better space utilization. The project team was responsible to come up with the newer draft of the plan and sharing it with all the participants over a week.

### Phase 2

The revised plan was reviewed by the participating team followed by a visit to the proposed PICU site. Participants in this phase were a clinical team of senior doctors and senior nurses, the project team, and the hospital administrators. Floor tapes of all the proposed bed spaces and other allocations were applied as per the new draft of the floor plan. During the simulation exercise, challenges in the location of the bed spaces and their orientation, nursing station, and the clean/dirty utility room were identified, and a revision of the plan was planned accordingly. The project team prepared a further revised plan draft and shared it with the participants over a week. A further simulated drill was conducted to ensure the acceptability of the revised plan. After consensus, the revised plan was agreed upon for implementation. The construction of an 8-bedded PICU was planned accordingly over the next couple of months.

### Phase 3

**FIGURE 1:**
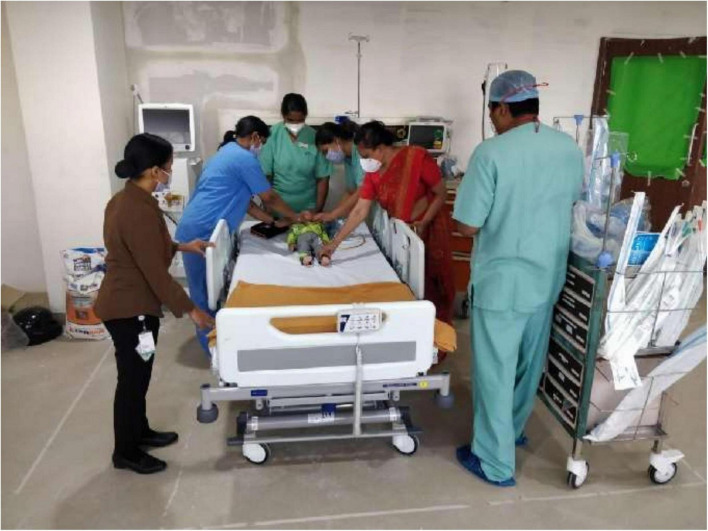
Phase 3 Simulation exercise with the placement of actual equipment and staff movement and bed space marked by floor tapes.

During phase 3 of simulation, the proposed PICU area was furnished with beds, monitors, ventilators, and infusion pumps as if a functional PICU over the 8-bed spaces. The clinical team of doctors and nurses, senior members of the project team, and the administrators participated in this exercise (Figure 1). Gaps were identified in the functionality of the planned 8-bedded space and following further simulation exercise to check the functionality, and there was consensus to make it a 6-bedded PICU design to accommodate adult-sized beds that will facilitate a comfortable mobilization and functionality. It was finally agreed to open the PICU as a 6-bedded PICU, and the unit was handed over for operationality to the clinical team by the project team.

### Phase 4

**FIGURE 2:**
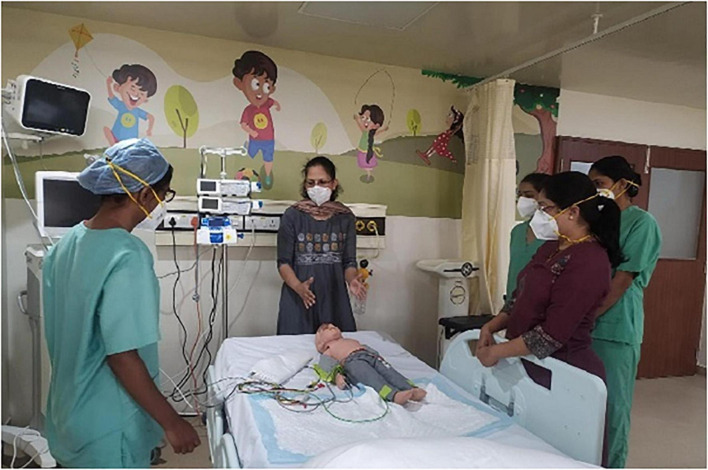
Phase 4 Simulation in-situ team training in the PICU.

During phase 4, the clinical team comprising of newly appointed doctors and nurses, and administrators participated in the *in situ* simulated exercises to review the preparedness for accepting patients in the newly setup clinical area (Figure 2). Induction and orientation of the environment were done for all the new team members as per department and hospital induction policies. Simulation scenarios included 5 themes – (i) Patient flow – reviewing the process of admission, paperwork trail, electronic health recording, documentation, handover between professionals, etc. (ii) Emergencies in PICU – code blue, airway emergencies, cardio pulmonary resuscitation, (iii) PICU procedures – setting up for invasive and non-invasive monitoring, intubation, central line, arterial line placement, ventilator setup and checks, etc. (iv) Communication – overhead speakers, telephone lines, and counseling room, and (v) operational issues – billing, security, fire, etc.

In total, five *in situ* simulated scenarios were conducted with various team combinations of the existent clinical team both during the daytime and night time to identify as many systems and team issues. Each simulated session identified gaps that had clear action points and the clinical lead worked with the PICU team to implement to close the gaps. The main gaps identified were situational awareness in the new environment, familiarization of team members to each other and other professionals within the hospital, familiarization of the available equipment or drugs, process flow, human factors, and crisis resource management. Cognitive aids were created and implemented for the ease of use. A video orientation of the unit depicting the placement of the equipment, drugs, and bed space was done and shared with the clinical team. Progressive bridging of the gaps occurred during the 5th simulation session, and there was a consensus of all the team members to open for patient care during the week of opening the PICU. Subsequently, work-as-simulated was matched with work-as-done.

## Discussion

This study describes the conduct of low-cost *in situ* stimulated exercises in a phasic manner involving an inter-professional team during the design of a PICU in the Indian context. The pre-construction 2 phases demonstrated the application of low-cost measures, such as floor tapes, to identify the gaps in the floor plans and effective utilization of the space. Following the debriefing and consensus, there was a re-design of the PICU plan and subsequent implementation. The post-construction phase 3 with the *in situ* simulation of the PICU space with all the equipment identified key mobility issues toward patient safety and hence a further plan of a 6-bedded PICU. Phase 4 of thematic *in situ* simulation identified system and process gaps, facilitated team training, and improvement in human factors. Implementation of safety measures enabled a consensus in the opening up of the PICU for patient care.

This study highlights the importance of understanding and improving human work as described by Shorrock (7–9) about the 9 proxies of work-as-done, such as work-as-imagined, work-as-prescribed, work-as-disclosed, work-as-analyzed, work-as-observed, work-as-simulated, work-as-instructed, work-as-measured, and work-as-judged, especially in the context of designing PICU in health care. There is a need to explore each of these aspects in a systematic manner. The participation of the clinical teams, inter-professional teams, and end users in an *in situ* simulated exercise is a key to addressing the gaps in safety and service as described in this study (9–11).

A process called SIMtest (3) that was applied in the design of a 24-bedded PICU has been reported at Barcelona describing the importance of involving the frontline professionals who are familiar with their work in the designing of the PICU from the beginning to finish facilitating a better workplace and safer patient processes for clinical care. A similar attempt has been made in this study where clinicians were involved from the start of the setting up of the PICU. The simulated exercises were planned in such a way that there was a consensus on the action plans that required implementation in a step-wise manner. The changes made following each of the simulation sessions indicate the benefit of ensuring the safety of patient care without imposing any harm to actual patients. However, the extensive input from other professionals, such as computer graphics experts, artificial intelligence, virtual hospital systems, human factor specialists, and quality managers, was limited in this study due to non-availability. However, the simulation exercise was conducted in a psychologically safe environment that achieved the consensus in implementing the changes following each of the exercises focusing on patient safety.

The low-cost resources such as floor tapes, low-tech manikins, and used and sterilized consumables are unique in this study that enable the simulation exercises to be conducted in a cost-effective way. Each session had lessons learned, and the changes could be implemented after consensus. The low-cost simulation resources are outlined mainly for task training traditionally (12). There are reports of cardboard simulations to simulate patient care areas that can be easily constructed and deconstructed with ease but are laborious (13). The methods applied in this study for *in situ* simulation with low-cost resources are routine practices for the authors. The cost-benefit analysis of such a phased simulation exercise is not performed in this study. However, it is expected that the application of the lessons learned and the further modifications would be in the best interest of the patient safety, and likely cost-effective.

The key challenges of implementing simulation exercises to design a patient care area appear to be the inter-professional coordination and buy-in, openness to revising plans, and focus on patient safety. The exact number of simulation sessions and the time frame required to achieve confidence and competence is not known. The designing of an acute patient care area would be easier during the initial construction phase of the entire building than designing the same as an add-on clinical area within the existent building. It appears that inter-professional simulation drills for such situations would be more valuable in ensuring adequate planning and implementation.

The applicability of similar simulation exercises in designing other patient care areas apart from the PICU needs to be studied further. There is a need for mutual respect and understanding while conducting inter-professional simulation, and hence, the replicability of this study in designing a PICU in other centers needs to be explored further. There is a need for guidelines to facilitate the conduct of a cost-effective simulation in a psychologically safe environment and debriefing strategies during the designing of patient care areas within the hospital.

## Conclusion

A focused and phased simulation exercise with low-cost resources involving the clinical and non-clinical team from the start to finish of the designing of the PICU is possible. Inter-professional simulation can enable the identification of structural challenges, design issues, latent safety threats, test systems, processes, patient flow, and facilitated team training during the design of a new PICU. Further studies are needed to understand the generalization of the study findings into designing PICU. Further guidance is required to understand the effective conduct of inter-professional simulation focusing on patient safety in designing patient care areas.

## Data availability statement

The raw data supporting the conclusions of this article will be made available by the authors, without undue reservation.

## Ethics statement

Ethical review and approval was not required for the study on human participants in accordance with the local legislation and institutional requirements. The patients/participants provided their written informed consent to participate in this study. Written informed consent was obtained from the individual(s) for the publication of any potentially identifiable images included in this article.

## Author contributions

ST conceived the idea and drafted the manuscript. ST, SG, CG, SA, and RC contributed to the simulation exercise. All authors contributed to the article and approved the submitted version.
